# Assessment of Implementation of Antimicrobial Resistance Surveillance and Antimicrobial Stewardship Programs in Tanzanian Health Facilities a Year After Launch of the National Action Plan

**DOI:** 10.3389/fpubh.2020.00454

**Published:** 2020-08-27

**Authors:** Raphael Z. Sangeda, Joel Kibona, Castory Munishi, Frank Arabi, Vicky P. Manyanga, Kennedy D. Mwambete, Pius G. Horumpende

**Affiliations:** ^1^Department Pharmaceutical Microbiology, Muhimbili University of Health and Allied Sciences, Dar es Salaam, Tanzania; ^2^Department of Medicinal Chemistry, Muhimbili University of Health and Allied Sciences, Dar es Salaam, Tanzania; ^3^Department of Biochemistry and Molecular Biology, Kilimanjaro Christian Medical University College, Moshi, Tanzania; ^4^Department of Microbiology, Immunology and Molecular Biology, Kilimanjaro Clinical Research Institute (KCRI), Moshi, Tanzania; ^5^Department of Preventive Medicine and Research, Lugalo General Military Hospital (GMH) and Military College of Medical Sciences (MCMS), Dar es Salaam, Tanzania

**Keywords:** antimicrobial resistance, antimicrobial stewardship, surveillance, national action plan, Tanzania

## Abstract

**Introduction:** Antimicrobial resistance (AMR) is a current global health threat and a challenge to the treatment of infectious diseases. The WHO advocates a strategy of antibiotic stewardship programs (ASP) in optimizing antimicrobial use in hospitals. This study aimed at assessing the existence of AMR surveillance and ASP implementation in health facilities in Tanzania in the year following the launch of the National Action Plan (NAP).

**Methodology:** From December 2017 through July 2018, a descriptive cross-sectional study was conducted using a structured questionnaire administered online. A total of 199 health facilities in Tanzania mainland whose contacts was obtained from the Ministry of Health Community Development Gender Elderly and Children (MoHCDGEC) were reached by phone and thereafter, a survey was sent via text or e-mail to focal persons in the corresponding facilities.

**Results:** Only 39 (32.5%) responses from contacted facilities were received and analyzed. Thirty (76.9%) of the facilities were government-owned. Of the 39 respondents surveyed, 13 (35.9%) declared to have implemented some sort of coordinated ASP to promote the rational use of antimicrobials at their facilities. The respondents reported the presence of guidelines for the implementation of ASP at variable proportions, whereas the presence of a committee for Infection Prevention and Control was reported by 27 (69.2%). Twenty-four (61.5%) had a Medical and Therapeutic Committee. Although all 39 (100%) respondents were aware of the presence of AMR in Tanzania, only 26 (66.7%) were aware of the presence of the Tanzanian NAP for AMR. Hospital antibiotic policy document was present in 6 (15.4%) facilities. Only 7 (17.9%) facilities conducted prescription auditing; 9 (23.1%) had a hospital formulary; 14 (35.9%) had standard hospital prescription. 9 (23.1%) had software for data storage about AMR. Only 7 (17.9%) facilities conducted microorganisms' susceptibility tests and kept the record of the microorganism susceptibility testing.

**Conclusion:** Our study found the existence of AMR surveillance activities and ASP implementation in Tanzania, albeit at a low level. The implementation was inconsistent across the surveyed facilities. These data have identified areas of improvement in addressing AMR in Tanzania through the NAP.

## Introduction

Antimicrobial resistance (AMR) is a threat to global health as it causes increased mortality, morbidity, and high fiscal costs (1, 2). Overuse of antimicrobials as a result of over-prescription and the practice of empirical treatment has significantly contributed to the AMR (3, 4). Resources for rapid and accurate diagnosis of the causative pathogens lack in resource-limited settings. This leads to a preference for empirical antimicrobial therapy to treat infectious disease (5, 6) with consequent excessive and irrational antimicrobial consumption. Antimicrobial stewardship programs (ASP), including AMR surveillance, offer an opportunity to reduce excessive and inappropriate antimicrobial consumption and hence mitigate AMR.

On 28^th^ April 2017, Tanzania launched its National Action Plan (NAP) on Antimicrobial Resistance 2017–2022 in response to the Agenda of the 68^th^ World Health Assembly (WHA) in May 2015. According to the Assembly resolutions, optimization of the use of antimicrobials was one of five pillars suggested (7) to combat AMR. This optimization is possible only through the establishment of hospital antimicrobial stewardship programs (ASP). ASP is the orderly approach to ensure the appropriate use of antimicrobials through promoting the selection of the optimal antimicrobials to the right patient, correct diagnosis, correct disease condition, right dose, strength, duration, and route of administration (8, 9). It is crucial to strengthen the ASP for evidence-based prescribing to ensure rational antimicrobial use (10, 11). The Tanzanian Ministry of Health is currently implementing the NAP with the support of other stakeholders.

One key component of ASP is the surveillance of AMR (12). Surveillance is performed through laboratory testing of microbial isolates (13). Antimicrobial susceptibility testing provides local data, which is important in guiding the empirical treatment in resource-limited settings (14–16). The implementation of AMR surveillance and ASP in health care facilities is determined by the use of guidelines and tools available plus the activities aimed at attaining rational antimicrobial prescriptions (17).

In Sub-Saharan Africa, there is a paucity of AMR data due to a lack of surveillance and ASP implementation (18). Such data are important in monitoring and evaluating the implementation of the NAP on AMR. This study, therefore, aimed to investigate the presence of ASP and IPC programs at a health facility level, availability of surveillance tools and the proportion of facilities that implement AMR surveillance in Tanzania mainland in the year following the launch of NAP. The findings from this study may provide baseline data for the stakeholders implementing the Tanzanian NAP 2017–2022 on Antimicrobial Resistance to help in improving the rational use of antimicrobials and the reduction of AMR.

## Materials and Methods

### Study Design

This was a descriptive cross-sectional study utilizing a structured questionnaire administered to healthcare personnel in health facilities to assess the implementation of the current AMR surveillance and ASP in Tanzanian health facilities. The survey invitations were sent by text or e-mailed to focal persons in their respective facilities after the initial contact was made through mobile telephones.

### Study Area

The study was conducted in Tanzania mainland health facilities.

### Sampling Method and Study Participants

A convenient sampling method was used to obtain the facilities to be included. A list of all registered health facilities from the district to a national level was obtained from the Ministry of Health Community Development Gender Elderly and Children (MoHCDGEC). A convenient sample size of 199 participants in 134 districts healthcare facilities in Tanzania mainland who had phone numbers registered were identified. Of these, a total of 120 were reached by phone, consenting process was initiated and contacts provided means they would prefer to receive the link to the online survey.

The study participants were representative of the health facilities who were pharmacists, doctors, or laboratory scientists. The study participants were interviewed using a structured questionnaire that contained questions relevant to the levels of implementation of ASP and antimicrobial resistance surveillance.

### Study Period

The survey was conducted over seven months from December 2017 through July 2018.

#### Data Collection Techniques

Data collection was done by initial contact through mobile phones to invite participants into the study. Upon the agreement to participate, the written consent letter and an online semi-structured questionnaire were sent through an SMS text, WhatsApp, or e-mail. Detailed information on how to respond to the survey was also sent to the participants. The online questionnaire investigated the implementation of ASP and AMR surveillance activities in the health care facilities from the district to the national level. We further identified the presence of the Hospital Therapeutic Committee (HTC), infection prevention and control (IPC) guidelines, hospital IPC committee, the use of standard prescriptions, the presence and use of national treatment guidelines, the presence and use of hospital antibiogram, reports and audit of antimicrobial use, and hospital antibiotic policy document.

#### Data Management and Analysis

The survey was created online and managed using Research Electronic Data Capture (REDCap) platform for data collection hosted at Muhimbili University of Health and Allied Science. REDCap is a secure, web-based application designed to support data capture for research studies, providing (1) an intuitive interface for validated data entry; (2) audit trails for tracking data manipulation and export procedures; (3) automated export procedures for seamless data downloads to standard statistical packages; and (4) procedures for importing data from external sources (19, 20). All data collected were counter-checked for their validity and clarity by removing redundant data. The coded data were analyzed using Statistical Package for Social Sciences (SPSS) version 23 computer analysis software. Frequencies of different ASP activities were described in tabular or graphical format.

#### Ethical Considerations

Ethical clearance to conduct this study was obtained from Muhimbili University of Health and Allied Sciences Research and Publications Committee with reference number DA. 25/111/01. Written informed consent was signed electronically before filling the questionnaire after a thorough explanation of the study objectives. The respondents were informed to have the freedom to reject participation in the study. The participants' names were not recorded (only codes were used) and all other personal information was handled with confidentiality throughout data collection.

## Results

### General Description of the Participated Health Care Facilities

**Figure 1:**
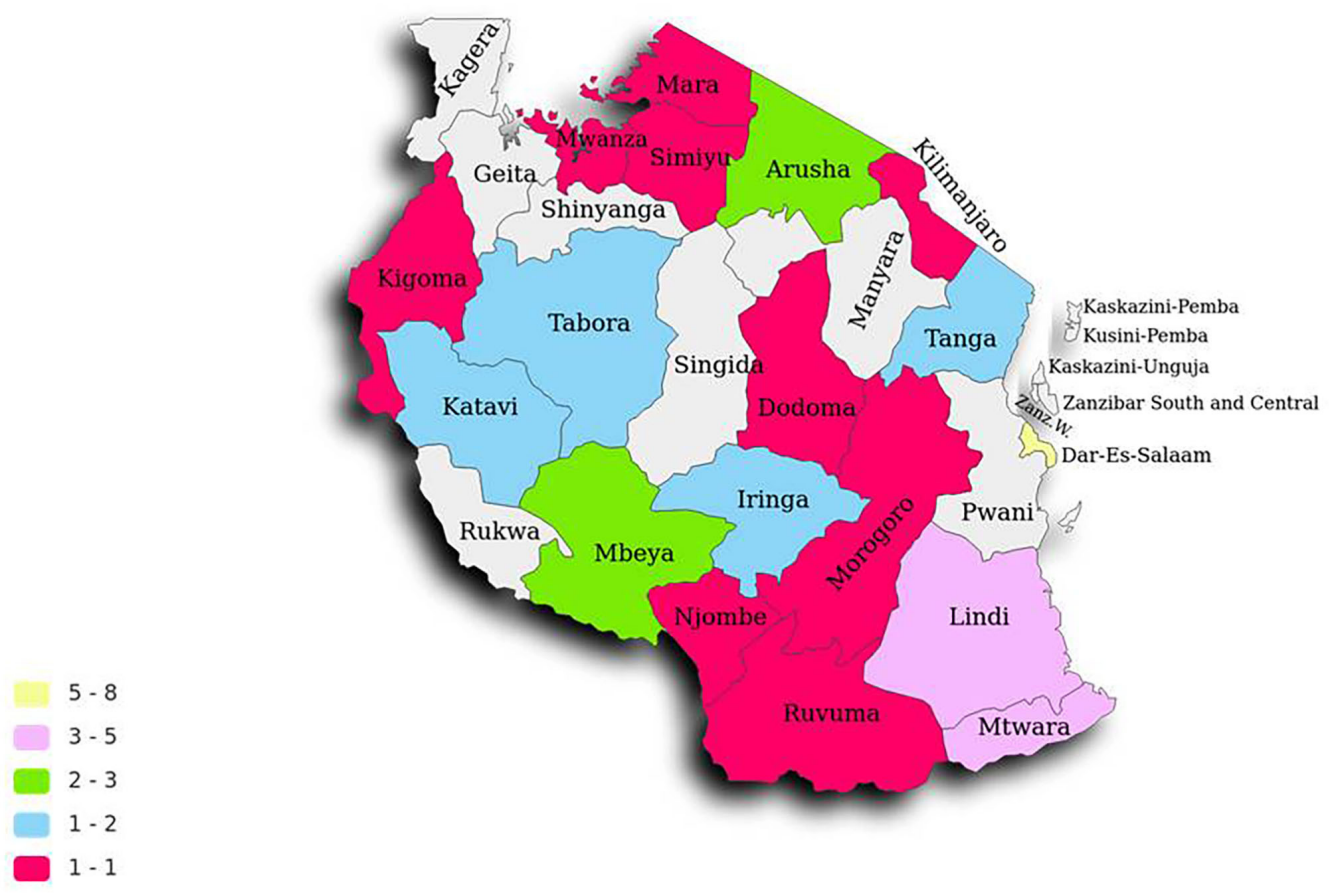
Geopolitical distribution of respondents in Tanzania mainland regions' facilities.

A total of 44 participants attempted to fill the survey; during data cleaning, five incomplete records were removed. Only 39 responses were further analyzed. The respondents in these facilities had median working experience 4.9; interquartile range 2.0–7.5 years. The responses were recorded in 19 geopolitical regions of mainland Tanzania (Figure 1). The majority of respondents were clinical pharmacists 30 (76.9%), followed by pharmaceutical technicians 4 (10.3%) and medical doctors 3 (7.7%). The majority of surveyed facilities were government health facilities 30 (76.9%); the rest were privately owned. Most of the facilities from which respondents came were at district level 15 (38.5%) followed by regional levels 11 (28.2%) (Table 1).

**Table 1:** Respondents personal and health facility characteristics.

**Characteristic**		**Frequency**	**Percent**
**Job position at the facility**			
	Pharmacist	30	76.9
	Pharmaceutical technician	4	10.3
	Medical doctor	3	7.7
	Assistant medical officer	1	2.6
	Biomedical researcher	1	2.6
**Facility Sponsorship type**			
	Private	9	23.1
	Government	30	76.9
**Region of the facility location in Tanzania**			
	Dar es Salaam	7	17.9
	Lindi	4	10.3
	Mtwara	4	10.3
	Arusha	3	7.7
	Iringa	2	5.1
	Katavi	2	5.1
	Mbeya	2	5.1
	Tabora	2	5.1
	Tanga	2	5.1
	Dodoma	1	2.6
	Kigoma	1	2.6
	Kilimanjaro	1	2.6
	Mara	1	2.6
	Morogoro	1	2.6
	Mwanza	1	2.6
	Njombe	1	2.6
	Ruvuma	1	2.6
	Simiyu	1	2.6
	Songwe	1	2.6
	Not mentioned	1	2.6
**Level of Healthcare facility**			
	National level	5	12.8
	Zonal level	4	10.3
	Regional level	11	28.2
	District level	15	38.5
	Health center	4	10.3

### Antimicrobial Stewardship Implementation

In this study, all the 39 (100%) healthcare personnel that participated were aware of AMR. Thirteen (33.3%) were aware of the 2017–2022 Tanzanian National Action Plan (NAP) on Antimicrobial Resistance. A total of 14 facilities (35.9%) had a committee dealing with AMR. The majority of the personnel in AMR committees were pharmacists and medical doctors, while the rest were nurses and clinical microbiologists. Nine (23.1%) facilities had antimicrobial use reports (Table 2).

**Table 2:** The level of awareness and implementation of antimicrobial stewardship activities.

**Characteristic**		**Frequency**	**Percent**
**Awareness of presence of AMR in Tanzania**			
	Yes	39	100.0
	No	0	0
**Awareness of presence of The NAP on AMR 2017–2022 in Tanzania**			
	No	13	33.3
	Yes	26	66.7
**Presence of an ASP committee on AMR in the facility**			
	No	25	64.1
	Yes	14	35.9
Does the AMR committee have either of the following?^*^			
	Medical doctor	9	23.1
	Pharmacist	10	25.6
	Medical laboratory scientist	8	20.5
	Pharmacologist	1	2.6
	Microbiologist	4	10.3
	Nurse	6	15.4
	Environmental scientist	3	7.7
	Pathologist	1	2.6
**AMR awareness and education to health workers at the facility**			
	No	12	30.8
	Yes	27	69.2
**Presence of Antimicrobial resistance levels/surveillance report at**			
**your facility**			
	No	32	82.1
	Yes	7	17.9
Organisms under AMR surveillance at your facility^*^			
	*Escherichia coli*	6	15.4
	*Staphylococcus aureus*	4	10.3
	*Klebsiella pneumonia*	3	7.7
	*Acinetobacter baumannii*	0	0.0
	*Pseudomonas aeruginosa*	2	5.1
	*Enterococci species*	1	2.6
	*Clostridium dificcile*	1	2.6
	*Candida species*	1	2.6
**Presence of a software to record antimicrobial susceptibility results**			
	No	30	76.9
	Yes	9	23.1
**Presence of any antimicrobial use report in the facility**			
	No	30	76.9
	Yes	9	23.1
**Where do you share/send the feedback or report on**			
Antimicrobial use?^*^			
	District medical officer	5	12.8
	Regional medical officer	2	5.1
	Ministry of health	2	5.1
	Health care providers at the health center	1	2.6
	Medical director of the hospital	1	2.6
	Therapeutic committee	1	2.6

* *, Multiple responses were available for the question; ASP, antimicrobial stewardship program; AMR, antimicrobial resistance; NAP, national action plan*.

### Infection Prevention and Control Practice

**Figure 2:**
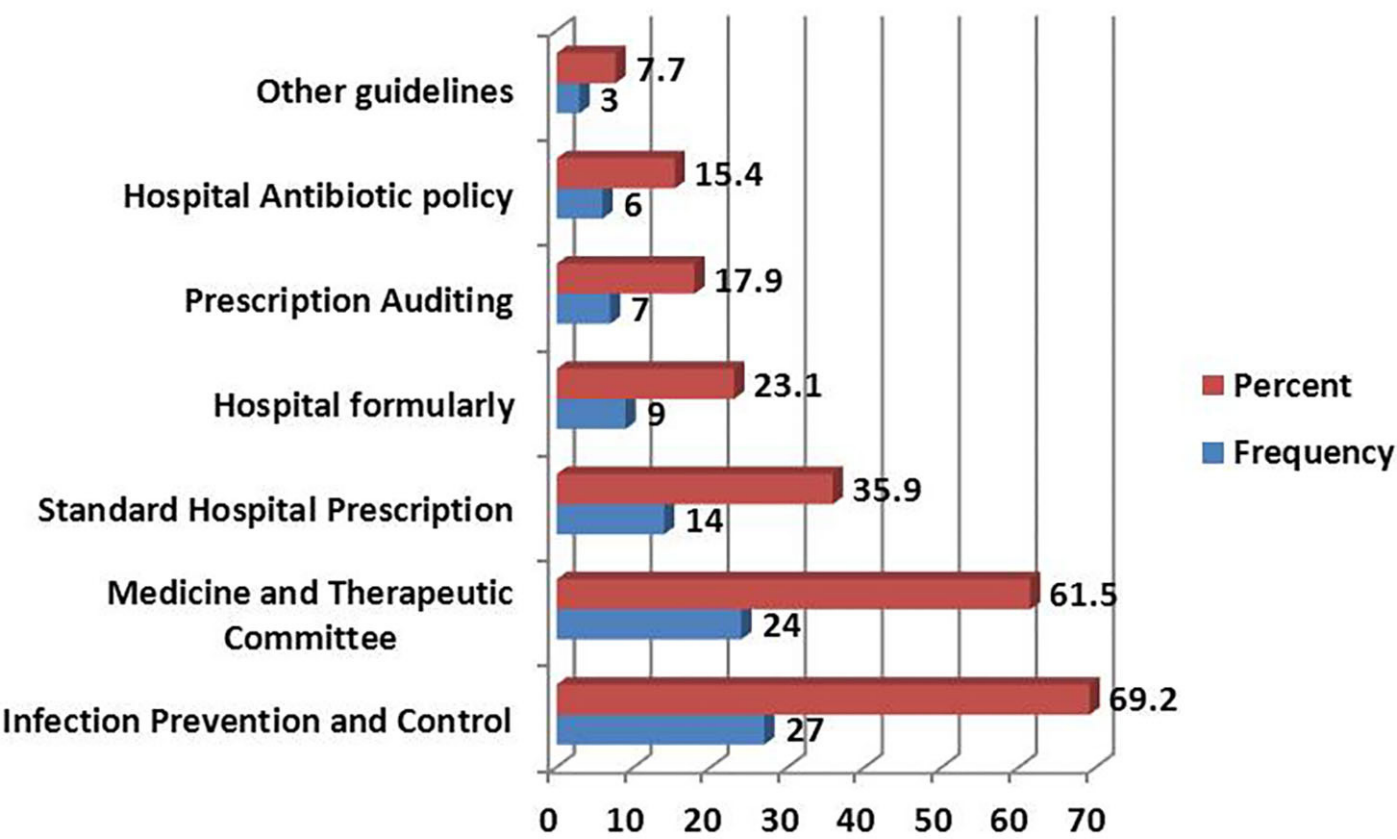
Type of guidelines used at the health facilities.

**Figure 3:**
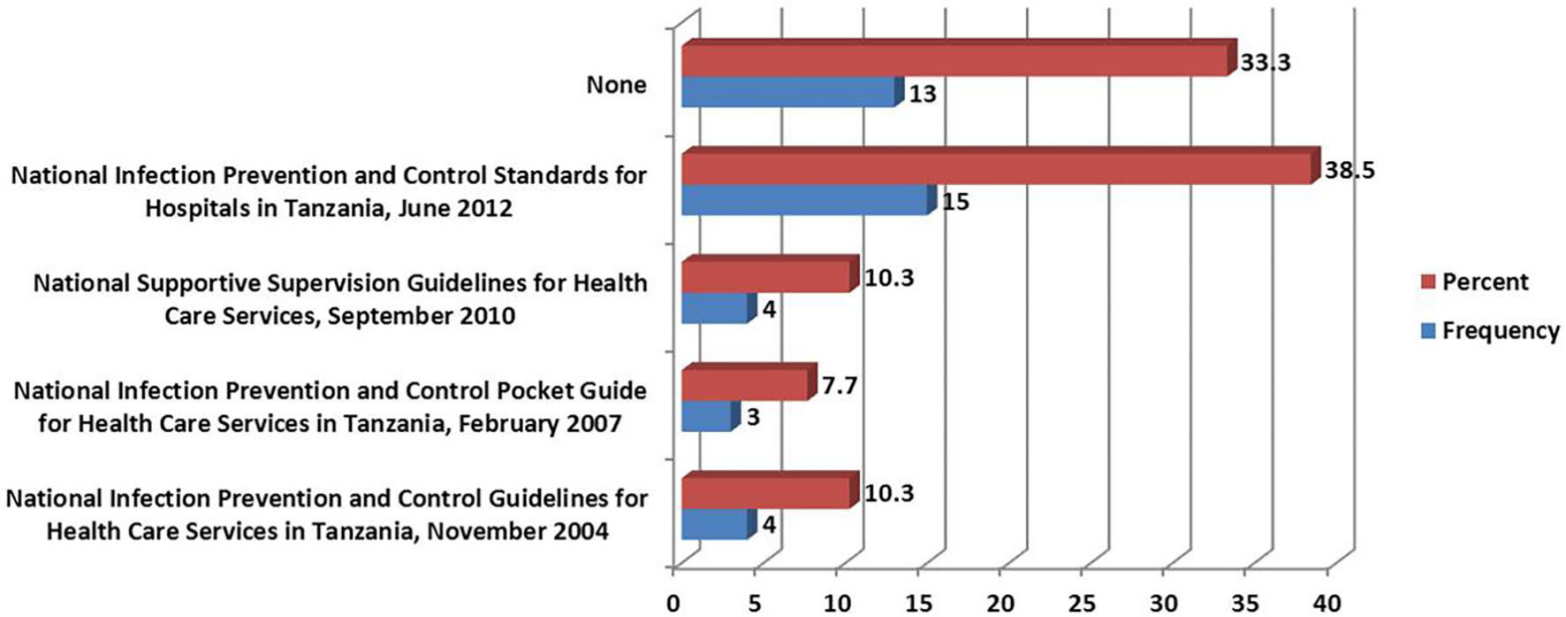
Versions of infection prevention and control guidelines used at the health facilities.

The most-reported guidelines present in the facilities were those relating to Infection Prevention and Control 27 (69.2%). Facilities with the Medical Therapeutic Committee guidelines were 24 (61.5%). The least reported guidelines in the facilities were on Hospital antibiotic policy, where only six facilities (15.4%) possessed them (Figure 2). The commonly used version for IPC guidelines was that of the National Infection Prevention and Control Standards for Hospitals in Tanzania of June 2012, where 15 (38.5%) facilities had the document while 12 (30.8%) facilities did not have the document at all (Figure 3).

### Use of the Standard Treatment Guidelines

**Figure 4:**
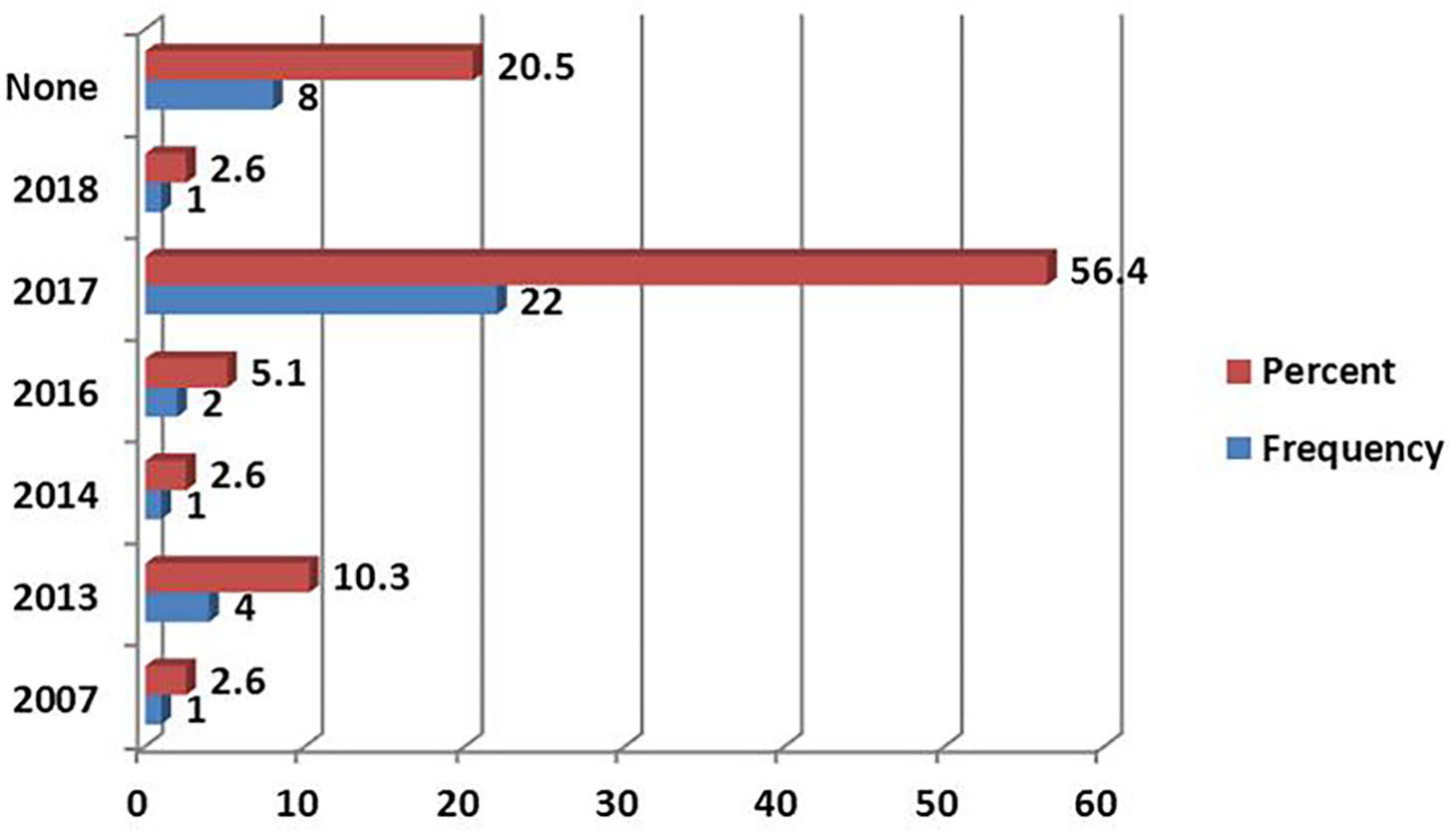
Versions of Standard treatment Guidelines (STG) used at the health facilities.

**Figure 5:**
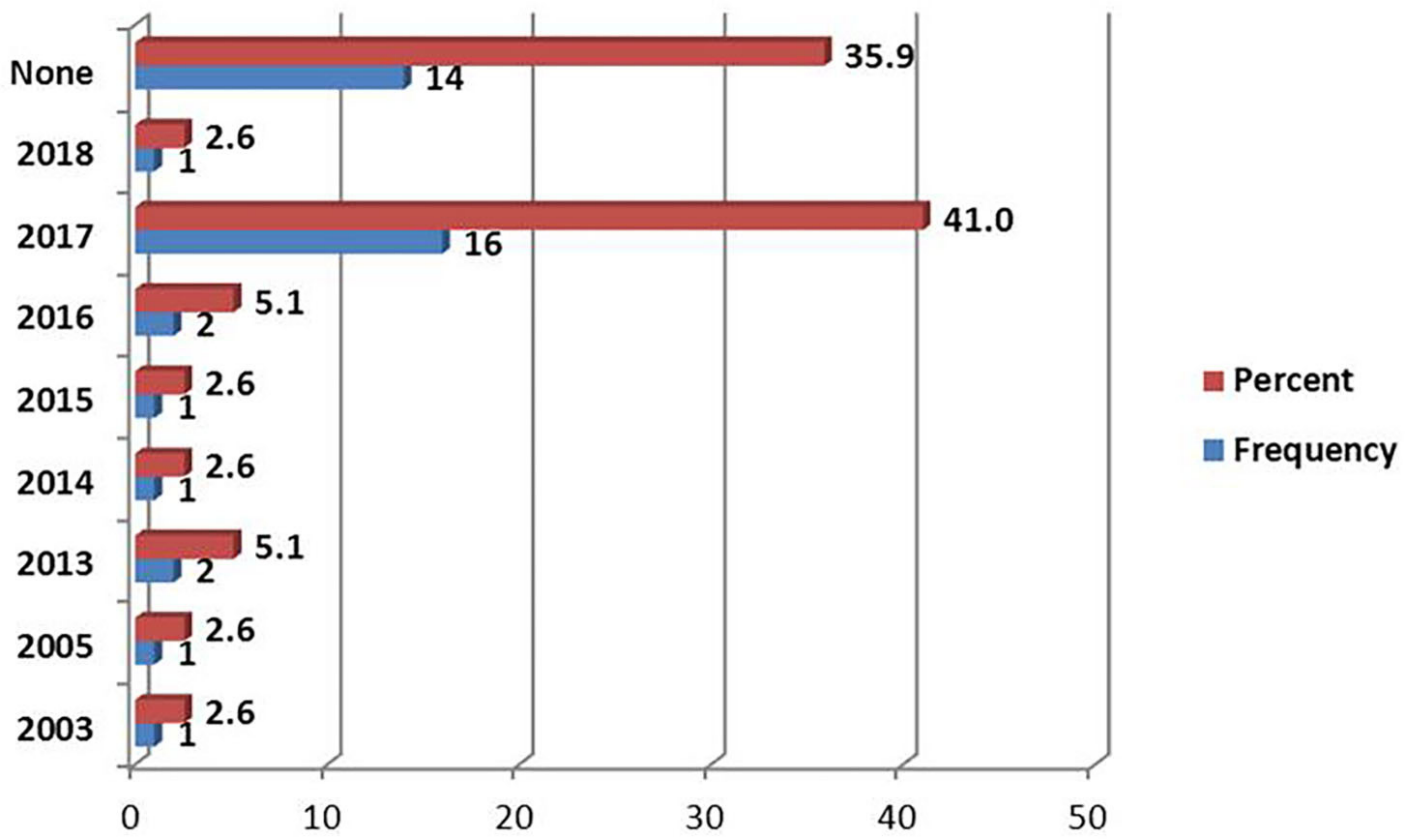
Versions of National Essential Medicine Lists (NEMLIT) used at the health facilities.

The 2017 Standard treatment Guideline (STG) was the most reported version document, followed by 2013 and 2016 (Figure 4). The most available National Essential Medicine List (NEMLIT) was of the year 2017, which was reported by 16 (41%) of respondents (Figure 5).

### AMR Awareness and Education to Health Workers

AMR awareness and education to health workers' activities were conducted in 27 (69.2%) of the health facilities (Table 2).

### Status of the Surveillance of Antimicrobial Susceptibility Testing

Of the surveyed facilities, 7 (17.9%) reported keeping a surveillance report following antimicrobial susceptibility testing. The most frequent organisms for which reports of antimicrobial susceptibility were recorded included *Escherichia coli–*6 (15.4%), *Staphylococcus aureus–*4 (10.3%) and *Klebsiella pneumonia–*3 (7.7%). None of the facilities reported to conduct or report antimicrobial susceptibility testing for *Acinetobacter baumannii*. Nine facilities (23.1%) had software to record the profile of antimicrobial susceptibility test results for specific microorganisms. Reporting and maintenance of records on antimicrobial use (AMU) occurred in 9 (23.1%) of the facilities. Only 5 (12.8%) of the reports are reportedly sent from the facilities to higher authorities at the district hospital level and much less 2 (5.1%) to the Ministry of Health (Table 2).

## Discussion

The study results highlight the current state and progress of ongoing implementation NAP on AMR in terms of antimicrobial stewardship programs (ASP) activities in health care facilities in mainland Tanzania. Our results give an insight into areas of improvement at the facility level that need the attention of the NAP. Overall, our study found the existence of AMR surveillance activities and ASP implementation in Tanzania, albeit at a low level. The implementation of ASP was inconsistent across the surveyed facilities.

The sample included mostly pharmacists and medical officers of respective facilities. We also asked them to refer the survey to relevant stakeholders in their facilities. The fact that the majority of pharmacists rather than medical officers responded to our survey may be an indication that pharmacists are taking active roles in stewardship activities in these facilities.

The study confirmed the availability of guideline tools for running the ASP with most of the facilities possessing the latest versions. These include the Standard Treatment guideline (STG), National Essential Medicine List (NEMLIT) and Infection Prevention and Control Guideline (IPC). Of these facilities, only 14 (35.9%) have the standard prescription, while 25 (64.1%) have no standard prescription used in their facilities.

There was a low practice of prescription auditing, where only a few facilities implement this activity. Among the 14 facilities with the standard prescription, only seven (17.9%) of them conduct the audit on the prescription to observe the trend of consumption, indication and quantities of the drugs.

A well-coordinated ASP promotes the appropriate use of antimicrobials, improves patient outcomes, reduces microbial resistance, and decreases the spread of infections, including hospital-acquired infections (HAI). In some cohort studies, the ASP proved to have higher survival rates and less Defined Daily Doses per 1,000 patient-days (21, 22).

In this survey in Tanzania, we tried to assess the implementation of some components for antimicrobial surveillance and ASP including the use of tools such as the Global AMR Surveillance System (GLASS) (23), among other activities.

As the science of antimicrobial stewardship advances, it is essential that well-conducted evaluations focused on patient and microbial outcomes are done to serve as the evidence base that directs optimal ASP intervention design and implementation. This study, therefore, provides guidance and recommendations for the design of studies to evaluate the impact of ASP interventions on the patient and microbial outcomes (8) in similar settings.

The implementation of antimicrobial stewardship programs and surveillance within hospitals is of high importance. These programs ensure effective monitoring of trends in antibiotic consumption, microbial infection, and microbial resistance against antibiotics. ASP is also important in developing drug policy and standard treatment guidelines that enhance best practices for patient care (24). In this study, 14 (35.9%) of surveyed health facilities have an AMR committee to implement the ASP. This is a small coverage considering that the Tanzanian National Action Plan for AMR was launched three years ago with all the necessary emphasis and a promise to act fast and efficiently (7). This indicates that there is an ineffective implementation of ASP in the first year, even though facilities claim to have some tools and information concerning the programs. This situation is a cause for concern in the fight against AMR and therefore needs to be improved.

In our study, only 14 (35.9%) facilities have the standard prescription, while 64.1% have no standard prescription used in their facilities. Moreover, only half of these facilities with standard prescription had the prescription auditing. This indicates poor practice in antimicrobial stewardship and surveillance on controlling the trend of use of antimicrobials and therefore calls for further enforcement of ASP strategies to improve the performance of the NAP.

The survey respondents report very few meetings of stewardship committees by the healthcare workers, even though this is an important component of ASP. Therefore, hospitals may have insufficient information on ASP management and operations. In the hospital environment, the prescribers and dispensers should have regular communication to iron out challenges in handling antimicrobials. The lack of such communication due to scarce meetings is equally another impediment in the fight against AMR in Tanzania.

There were only a few health facilities conducting susceptibility tests and recording the microorganism isolated 7 (17.6%). Moreover, only a few facilities possess software to keep a record for the tests and further analysis. Unavailability of the software to record the susceptibility test results of microorganisms in these health facilities leads to poor storage of data and uncoordinated systems. This is another factor that makes surveillance of resistant microorganisms and informed ASP implementation difficult to coordinate. The lack of susceptibility testing potentially leads to the empirical treatment with antibiotics, which ultimately leads to excessive and irrational antimicrobial use and consequently, to the emergency of AMR.

Concerning the coordination and reporting, our findings from the healthcare facilities show poor coordination of AMR activities. Lack of a formal surveillance system on AMR activities from different sites makes inconsistent data sharing to the higher levels. This reporting inconsistency impedes monitoring and evaluation (M&E) of AMR at the National level. Thus, there is a need to create a uniform reporting system from all sources of AMR data to coordinate the M&E.

## Conclusion

From our study, we conclude that there some antimicrobial stewardship program (ASP) implementation existing in Tanzania, with variation across facilities in geopolitical regions of Tanzania. The absence or minimal standard prescription and auditing, irregular, and few numbers of hospital committee meetings of the committees in carrying out the ASP and AMR surveillance programs, few sites performing antimicrobial susceptibility testing and data storage lead to ineffective implementation of the ASP and surveillance. There is no clarity on the flow of information and data from the facilities to the higher ranks in the system for further evaluation and monitoring.

## Recommendations

The formation of a formal AMR surveillance system is necessary for the coordination of activities. The government and private healthcare stakeholders should join in ensuring increased awareness and continued training to equip healthcare workers with the knowledge to implement ASP activities. The responsible ministry should ensure the availability of required tools to run the ASP in the health care facilities all over the country. Moreover, an extensive study conducted from a large number of health facilities to evaluate the three-yearly progress of the NAP implementation after its roll-out and escalation of ASP in the country is warranted.

## Limitation of the Study

Even though 120 participants in 134 districts of mainland Tanzania were invited to the online survey, only 39 (32.5%) from a few health facilities in the given regions were willing to participate. The low response rate is typical in other online surveys, in which case the one with active ASP would be more likely to respond. In a few studies that use an online survey in Tanzania, a small response rate has been noted among the invited participants. The online-based survey by Ruhwanya and Ophoff to Small Medium Enterprises (SMEs) members from the Tanzanian chamber of commerce industry and agriculture (TCCIA) sent 100 and 13 e-mails invitations but only 14 responses were received (25). In another study by Lwoga an online survey was distributed to 235 faculty members in five schools and one institute at Muhimbili University of Health and Allied Science, with a response rate of 34.5 percent (26). Other studies have shown that web-based survey has a lower response rate compared to the paper-based survey (27).

Consequently, a few regions' facilities were not represented in the results, thus limiting the ability to make a precise situational analysis of all hospitals in Tanzania regarding the implementation of IPC, AMR surveillance, and ASPs. Nevertheless, this study gives a snapshot situational analysis of the current state of the implementation of antimicrobial surveillance in Tanzania. As such, it serves as the baseline report of AMR surveillances and stewardship programs before full implementation of the National Action Plan on Antimicrobial Resistance 2017–2022 in Tanzania.

## Data Availability Statement

The raw data supporting the conclusions of this article will be made available by the authors, without undue reservation.

## Ethics Statement

Ethical clearance to conduct this study was obtained from Muhimbili University of Health and Allied Sciences Research and Publications Committee with reference number DA. 25/111/01. Written informed consent was signed electronically before filling the questionnaire after a thorough explanation of the study objectives. The respondents were informed to have the freedom to reject participation in the study. The participants' names were not recorded (only codes were used) and all other personal information was handled with confidentiality throughout data collection.

## Author Contributions

RS and JK conceived and supervised the work reported in this paper. PH, CM, and FA analyzed the data. All authors contributed to the review and discussions of the manuscript. RS and VM first drafted the paper. KM and PH comprehensively reviewed the manuscript. All authors read and commented on drafts and approved the final manuscript.

## Conflict of Interest

The authors declare that the research was conducted in the absence of any commercial or financial relationships that could be construed as a potential conflict of interest.
