# Supplementary material for: Assessment of Implementation of Antimicrobial Resistance Surveillance and Antimicrobial Stewardship Programs in Tanzanian Health Facilities a Year After Launch of the National Action Plan
Source: Front Public Health. 2020 Aug 27;8:454. doi: 10.3389/fpubh.2020.00454 (PMC7481440; doi:10.3389/fpubh.2020.00454)
Supplement: Supplementary file 1 [file Data_Sheet_1.docx]

**Supplementary Table**

Supplementary Table 1: Regional distribution of health facility surveyed by type of facility sponsorship

| **Region** | **Private** | **Government** | **Region Total** |
| --- | --- | --- | --- |
| Dar es Salaam | 2 | 5 | 7 |
| Lindi |  | 4 | 4 |
| Mtwara | 1 | 3 | 4 |
| Arusha | 2 | 1 | 3 |
| Iringa |  | 2 | 2 |
| Katavi |  | 2 | 2 |
| Mbeya | 2 |  | 2 |
| Tabora |  | 2 | 2 |
| Tanga |  | 2 | 2 |
| Dodoma |  | 1 | 1 |
| Kigoma |  | 1 | 1 |
| Kilimanjaro |  | 1 | 1 |
| Mara |  | 1 | 1 |
| Morogoro | 1 |  | 1 |
| Mwanza | 1 |  | 1 |
| Njombe |  | 1 | 1 |
| Not Mentioned |  | 1 | 1 |
| Ruvuma |  | 1 | 1 |
| Simiyu |  | 1 | 1 |
| Songwe |  | 1 | 1 |
| **Type total** | **9** | **30** | **39** |

Supplementary Table 2: Regional distribution of health facility surveyed by level of facility

| **Region** | **District level** | **Regional level** | **Zonal level** | **National level** | **Other level** | **Region Total** |
| --- | --- | --- | --- | --- | --- | --- |
| Dar es Salaam |  | 1 | 1 | 5 |  | 7 |
| Mtwara | 3 | 1 |  |  |  | 4 |
| Lindi | 2 | 2 |  |  |  | 4 |
| Arusha |  |  | 1 |  | 2 | 3 |
| Katavi | 1 |  |  |  | 1 | 2 |
| Tabora | 1 | 1 |  |  |  | 2 |
| Tanga |  | 2 |  |  |  | 2 |
| Mbeya | 1 |  | 1 |  |  | 2 |
| Iringa | 2 |  |  |  |  | 2 |
| Songwe | 1 |  |  |  |  | 1 |
| Not Mentioned |  | 1 |  |  |  | 1 |
| Ruvuma |  | 1 |  |  |  | 1 |
| Kigoma | 1 |  |  |  |  | 1 |
| Dodoma |  | 1 |  |  |  | 1 |
| Njombe |  |  |  |  | 1 | 1 |
| Mara | 1 |  |  |  |  | 1 |
| Kilimanjaro | 1 |  |  |  |  | 1 |
| Simiyu |  | 1 |  |  |  | 1 |
| Mwanza |  |  | 1 |  |  | 1 |
| Morogoro | 1 |  |  |  |  | 1 |
| **Type total** | **15** | **11** | **4** | **5** | **4** | **39** |
